# Supplementary material for: Availability and readiness of health facilities for non-communicable disease services in Ethiopia: Evidence from the nationally representative health facility survey 2022
Source: PLoS One. 2025 Dec 5;20(12):e0336675. doi: 10.1371/journal.pone.0336675 (PMC12680257; doi:10.1371/journal.pone.0336675)
Supplement: S1 Table — (DOCX) [file pone.0336675.s001.docx]

Supplementary Table 1: Summary of tracer items of each domain and measurement procedure of
readiness score of health facilities offering different non-communicable diseases-related services

| **Diabetes-related services** | |  | **Calculation of score** | |
| --- | --- | --- | --- | --- |
| **Domains** | **Tracer Indicators** | **Measurement** | **Domain score** | **Total score** |
| Guidelines and staffing training | Guidelines for diagnosis and management of diabetes **(a1)** | Yes= 1  No= 0 | A=(a1+b1)/2*100 | (A+B+C+D)/4 |
|  | Staff trained in the diagnosis and management of diabetes **(b1)** | Yes= 1  No= 0 |  |  |
| Basic diagnostic | Blood glucose **(c1)** | Yes= 1  No = 0 | B=(c1+d1+e1)/3*100 |  |
|  | Urine glucose **(d1)** | Yes= 1  No = 0 |  |  |
|  | Urine protein **(e1)** | Yes= 1  No = 0 |  |  |
| Essential medicines and commodities | Metformin **(f1)** | Yes= 1  No = 0 | C=(f1+g1+h1+i1)/4*100 |  |
|  | Glibenclamide **(g1)** | Yes= 1  No = 0 |  |  |
|  | Insulin injections **(h1)** | Yes= 1  No = 0 |  |  |
|  | Injectable dextrose solution **(i1)** | Yes= 1  No = 0 |  |  |
| Basic equipment | Weight scale **(j1)** | Yes= 1  No = 0 | D=(j1+k1+l1)/3*100 |  |
|  | Height scale (height board/stadiometer) **(k1)** | Yes= 1  No = 0 |  |  |
|  | Blood pressure (BP) apparatus **(l1)** | Yes= 1  No = 0 |  |  |
| **Cardiovascular diseases (CVDs) related services** | | | **Calculation of score** | |
| **Domains** | **Indicators** | **Measurement** | **Domain score** | **Total score** |
| Guidelines and staffing training | Guidelines for diagnosis and management of CVDs **(a2)** | Yes= 1  No = 0 | A=(a2+b2)/2*100 | (A+B+C)/3 |
|  | Staff trained in the diagnosis and management of CVDs **(b2)** | Yes= 1  No = 0 |  |  |
| Essential medicines and commodities | Calcium channel blockers  (Amlodipine/Nifedipine) **(c2)** | Yes= 1  No = 0 | B=(c2+d2+e2+f2+g2)/5*100 |  |
|  | Diuretics (Thiazide, Furosemide) **(d1)** | Yes= 1  No = 0 |  |  |
|  | ACE inhibitors (Captopril/Enalapril) **(e1)** | Yes= 1  No = 0 |  |  |
|  | Beta blockers (atenolol) **(f2)** | Yes= 1  No = 0 |  |  |
|  | Aspirin **(g2)** | Yes= 1  No = 0 |  |  |
| Basic equipment | Stethoscope **(h2)** | Yes= 1  No = 0 | C=(h2+i2+j2+k2)/4*100 |  |
|  | Blood pressure (BP) apparatus **(i2)** | Yes= 1  No = 0 |  |  |
|  | Adult weighing scale **(j2)** | Yes= 1  No = 0 |  |  |
|  | Oxygen **(k2)** | Yes= 1  No = 0 |  |  |
| **Chronic Respiratory Diseases (CRDs) related services** | |  | **Calculation of score** | |
| **Domains** | **Indicators** | **Measurement** | **Domain score** | **Total score** |
| Guidelines and staffing training | Guidelines for diagnosis and management of CRDs **(a3)** | Yes= 1  No = 0 | A=(a3+b3)/2*100 | (A+B+C)/3 |
|  | Staff trained in the diagnosis and management of CRDs **(b3)** | Yes= 1  No = 0 |  |  |
| Essential medicines and commodities | Beclomethasone inhaler **(c3)** | Yes= 1  No = 0 | B=(c3+d3+e3+f3+g3)/5*100 |  |
|  | Salbutamol inhaler or tablets **(d3)** | Yes= 1  No = 0 |  |  |
|  | Prednisolone tablets **(e3)** | Yes= 1  No = 0 |  |  |
|  | Hydrocortisone **(f3)** | Yes= 1  No = 0 |  |  |
|  | Epinephrine injection **(g3)** | Yes= 1  No = 0 |  |  |
| Basic equipment | Stethoscope **(h3)** | Yes= 1  No = 0 | C=(h3+i3+j3+k3)/4*100 |  |
|  | Peak flow meter **(i3)** | Yes= 1  No = 0 |  |  |
|  | Spacers for inhalers **(j3)** | Yes= 1  No = 0 |  |  |
|  | Oxygen **(k3)** | Yes= 1  No = 0 |  |  |
| **Mental Health-related services** | | | **Calculation of score** | |
| **Domains** | **Indicators** | **Measurement** | **Domain score** | **Total score** |
| Guidelines and staffing training | Guidelines for diagnosis and management of MNS use disorder **(a4)** | Yes= 1  No = 0 | A=(a4+b4)/2*100 | (A+B)/2 |
|  | Staff trained in the diagnosis and management of MNS use disorder **(b4)** | Yes= 1  No = 0 |  |  |
| Essential medicines and commodities | Amitriptyline **(c4)** | Yes= 1  No = 0 | B=(c4+d4+e4+f4+g4+h4+i4+j4+k4)/9*100 |  |
|  | Fluoxetine **(d4)** | Yes= 1  No = 0 |  |  |
|  | Carbamazepine **(e4)** | Yes= 1  No = 0 |  |  |
|  | Phenobarbitone **(f4)** | Yes= 1  No = 0 |  |  |
|  | Sodium valproate **(g4)** | Yes= 1  No = 0 |  |  |
|  | Diazepam **(h4)** | Yes= 1  No = 0 |  |  |
|  | Chlorpromazine **(i4)** | Yes= 1  No = 0 |  |  |
|  | Phenytoin **(j4)** | Yes= 1  No = 0 |  |  |
|  | Haloperidol **(k4)** | Yes= 1  No = 0 |  |  |
